# Some tributes to research colleagues and other contributors to our knowledge about kuru

**DOI:** 10.1098/rstb.2008.4000

**Published:** 2008-11-27

**Authors:** Michael P. Alpers

**Affiliations:** Centre for International Health, ABCRCHealth Research Campus, Shenton Park, Curtin University, GPO Box U1987, Perth, WA 6845, Australia; MRC Prion Unit and Department of Neurodegenerative Disease, UCL Institute of Neurology, The National Hospital for Neurology and Neurosurgery, Queen SquareLondon WC1N 3BG, UK

At the meeting at the Royal Society on ‘The end of kuru: 50 years of research into an extraordinary disease’, we celebrated the impending end of the kuru epidemic and the achievements of many people who had worked on kuru over the last 50 years. Especially honoured were our two Nobel laureates, D. Carleton Gajdusek and Stanley Prusiner. It was wonderful that Carleton, in his 85th year, could attend. More than being the father of kuru research, he is the veritable guru of kuru. I am delighted to be able to offer here yet one more tribute to him. Nevertheless, there are many others whose achievements have illuminated the unfolding story of kuru and their contributions should not be forgotten. At the meeting, there were notice boards in the Marble Hall outside the Kohn Room where participants could put on display photographs, memorabilia, tributes and the like; those who had been invited to the meeting but could not attend were offered the same opportunity. I placed on the board my tribute to my colleague Clarence Joseph Gibbs Jr, entitled ‘In celebration of Joe Gibbs’, written to honour Joe at a ceremony at the National Institutes of Health (NIH) in Bethesda, Maryland soon after his death on 16 February 2001, aged 76 years. I include extracts of it here.

Joe and I were part of the Laboratory of Central Nervous System Studies, headed by Carleton Gajdusek, which was based within the National Institute of Neurological Diseases and Blindness on the main campus of the NIH. The primate facility was set up in the countryside on land provided by the US Wildlife Service, in the beautiful woods of Patuxent, where Joe and I carried on the daily grind of the first transmission experiments with chimpanzees. We had their care, comfort and illnesses to worry about, regular clinical examinations to perform, blood to be taken and frequent filming sessions to cajole them into. Here we had a small team, with its own dynamics and its own loyalties, more tightly knit than the wider Laboratory and more intense. This team was to a large extent independent and Joe was its unquestioned leader. Mike Sulima and Al Bacote were senior members of the team. When I first wrote down the word ‘kuru’ in my clinical notes after examining one of the sick chimpanzees, Joe and I discussed the implications of this for a few minutes and he went into action: Carleton came back from Papua New Guinea (PNG) by the next available flights. He grumbled at first, but not the next day after he had seen Daisey and Georgette; Daisey, in particular, looked uncannily like human kuru. Later in the year, when the first autopsy was being done, Elisabeth Beck came to see that Joe and I were doing it right. Elisabeth took the brain back with her to London. When, in early February 1966, we received Elisabeth's telegram saying that the pathology of the chimpanzee brain was indistinguishable from human kuru, Carleton, Joe and I were all in Bethesda. We divided the paper into three parts and wrote it in a day. With the help of Marion Poms it was typed and mailed by midnight—and published in *Nature* within two weeks (Gajdusek *et al.* 1966).

Joe was a delightful and upright man, and in the same vein a rigorous scientist who took great delight in his work. He was warm and blunt, and full of quips; we were always happy to grumble about the world together. He enjoyed being part of a family, whether of colleagues, friends or relations, and took great pleasure in other people's children. I am sorry that I did not spend more time with him fishing on the Bay. I am privileged and happy to praise and honour him now: briefly, with so much unsaid and taken as understood—as is fitting for Joe, who kept his heart warm within and never wore it on his sleeve.

Mike Sulima was Joe's right-hand man and Al Bacote the chief animal attendant. Their care of the animals was critical to the success of our work. The facilities at Patuxent were not grand but the animals were given detailed attention and tender care; they all had names and were regarded as part of the Patuxent family. When Georgette and then Daisey started acting strangely, before there were objective signs of kuru-like ataxia, it was Al who first picked it up. We spent a lot of time together in the company of Georgette and Daisey—and Joanna…and, indeed, all the other inoculated chimpanzees in the unit. Both Mike and Al were invited to the meeting in London but, regrettably, were unable to come.

Marion Poms was Carleton's secretary and the mother of all the staff of the Laboratory. Her efficiency, warmth and loyalty were essential to keeping the research programme going, both in the Bethesda labs and in support of the work out in the field—which was principally in PNG and Micronesia but by no means only there. Carleton was mostly away, all over the world, and much responsibility therefore devolved upon Marion. She was unfailingly helpful to all of us, and adept at dealing with all the manuscripts that landed on her desk for typing—as was essential in those days before word processing. Marion was still there to share the joy of Carleton's Nobel Prize before she retired. She died several years later.

Elisabeth Beck was a neuropathologist based at the Maudsley Hospital in South London in the department of Professor Peter Daniel. They were strong supporters of Carleton's research programme over many years. When kuru had been successfully transmitted we looked about for brain material from a patient who had died of Creutzfeldt–Jakob disease (CJD). In the United States this diagnosis was made only by neuropathologists so we enlisted the aid of our English colleagues to find a patient who had been diagnosed in life, from whom we might obtain appropriate specimens for inoculation. Peter and Elisabeth managed to achieve this and thereby accelerated our experimental programme (Gibbs *et al.* 1968). Elisabeth provided the pathological support for our work on kuru and CJD (Beck *et al.*^1966^,^1969^). I had the pleasure of working with her for 6 weeks at the end of 1967 learning some neuropathology. She was an elegant person of the old school, who had been forced to flee Berlin in the 1930s (see fig. 6 in Asher 2008). When she provided lunch at her home for you it was always a fine meal served in style. Soon after her retirement she moved to a nursing home, where she eventually died.

A tragedy occurred around the End of Kuru conference that remains a sad puzzle. Hilary King worked on the epidemiology of kuru with Richard Hornabrook (King 1975). During his time in PNG he made many fine photographs, which, as he explained to me several months before the conference, he was keen to show at the Royal Society. He went to a lot of trouble to prepare them. Though he was reluctant to speak at the conference, for reasons that were not clear to me, he eventually agreed to have his 5-minute slot on the first day like everybody else. At the last minute he called London to say that he could not attend because he was not well and asked whether his wife could come in his stead. This was readily agreed to and his wife Elena came and participated in the meeting. She displayed on the notice board a small collection of Hilary's superb photographs and his *Lancet* paper on kuru. When Elena returned home to France after the meeting, tragically she found Hilary dead in their house. No cause for his death was ascertained. Although Hilary and I interacted only occasionally when he was working in PNG on kuru, subsequently we became good friends and close collaborators on studies of diabetes, hypertension and an index of modernity in PNG. He became the head of the section on noncommunicable diseases (NCD) at the World Health Organization in Geneva. All his colleagues will mourn his death, so sadly associated with our conference, and honour his contributions to research on kuru. I would like personally also to acknowledge his important studies on NCD in PNG and, especially, his contributions to the control of NCD throughout the world, and I mourn the loss of a cherished friend.

Until the 1940s the people of the kuru-affected region lived a fully traditional life with no direct knowledge of the outside world, even though there were omens of change with planes of the Second World War flying over and, in a few cases, crashing into their ground. In 1949 a Lutheran Mission was set up at Tarabo on the edge of the kuru region. In the early 1950s the process of establishing administrative control over this as yet ‘uncontrolled’ region began. A police post was set up at Okapa in 1951, which became the government station in 1954 with a resident Australian patrol officer. Inevitably the early patrols into the region were confronted by the strange phenomenon of kuru, and their reports, such as that of John MacArthur in 1953, alerted the Australian Administration to this unusual problem. Census and other patrols by John Colman from the new station at Okapa brought in more information about the extent of kuru. Frank Earl, a European Medical Assistant (EMA), as a member of the patrol team, made percipient comments about kuru. The Administration responded to these reports by sending the Government Anthropologist, Charles Julius, to Okapa. He studied kuru at Wanitabi village in the South Fore and wrote an insightful account of it in his official report in early 1957. In 1957 the patrol officer at Okapa was Jack Baker. Kuru was clearly such a problem in his jurisdiction that he became an integral part of the research team of Carleton Gajdusek and Vin Zigas. He conducted frequent census patrols throughout the district and, in collaboration with Carleton, determined the boundaries of the kuru-affected region. The Administration again responded positively and initiated regular six-monthly census patrols in the district administered from Okapa, especially to establish the incidence and prevalence of kuru and the specific mortality from the disease. Even if they did not use these epidemiological terms, the patrols conducted by a succession of conscientious patrol officers, cadet patrol officers, EMAs and police provided, during the peak of the epidemic, the basic epidemiological information that was needed. This practice carried on beyond the time I was in Okapa, when the patrol officer was Mert Brightwell, and made the task of good epidemiological surveillance possible ([Bibr bib8Q2][Bibr bib1Q2]). I salute their individual and collective, and largely unsung, contributions to our knowledge about kuru. Of those mentioned by name, John Colman and Jack Baker were invited to the conference but regrettably were unable to attend.

When I decided, as a medical student, that I wanted to go to Okapa to work on kuru, I was supported in every way by Norrie Robson, the Professor of Medicine at the University of Adelaide. He had initiated the work of the Adelaide group on kuru and contributed to an important early paper on the clinical features of kuru (Simpson *et al.* 1959). He showed interest in my ideas that emerged from spending all the time I could in the Library devouring every paper that had been written about kuru and the people of the Okapa area—not many in those days, but there was plenty to chase up in their end references. He secured for me a Rockefeller Fellowship—which eventually I did not need since the Administration of the Territory of Papua and New Guinea decided to quarantine the kuru region to prevent the putative deadly gene of kuru from spreading (a policy that was never in the end carried out) and, in compensation, created a position for a research medical officer in Okapa, which I obtained through the auspices of Professor Robson. Moreover, he used his Rockefeller grant to buy me an excellent Bolex movie camera—which is still in use. I have many reasons to be grateful to Norrie Robson.

The hospital medical officer when I arrived in Okapa was Andrew Gray. Andrew was very helpful in settling me in and very solicitous of my well-being—and that of my family. He supported all my plans, such as to base my fieldwork in a village in the South Fore, when he might have preferred to have another doctor on the station. Previously he had contributed to the research studies of the Adelaide group, and he was equally supportive of the work of Carleton Gajdusek—despite the fact that certain authorities in Port Moresby had given him strict instructions to do all he could to thwart Carleton's activities! Andrew and I agreed about the value of Carleton's work—indeed about most things—and on occasion we combined forces to counteract government bureaucracy. We also agreed that kuru patients and the so-called kuru orphans should be cared for by their extended families in their home environment. The Lutheran Mission had obtained funds to build a hospital to care for kuru patients and children who had lost their mothers from kuru. This was built in Okapa. When Andrew as the doctor responsible for the hospital said that kuru patients would no longer be admitted unless they had some other condition requiring hospitalization, the newly built hospital was sold to the government and the Lutheran Mission built the Kuru Centre at Awande, about 3 km away. Patients and kuru ‘orphans’ were cared for there until the patients stopped coming. Later, when the local people realized that they did not have to put their children in the orphanage, the children were also taken home. Highland PNG societies were accustomed to adoption and capable of taking care of their own children who had lost one or both parents, since they were doing it all the time. However, patients often needed help with nursing care and children nutritional support, so, finally, a Lutheran Mission nurse based in Awande went out and provided for people's needs in their home environment. Andrew left Okapa before the denouement of this saga but I know he would have been pleased by its outcome. When he left he gave me his books, which I can recognize by the mustiness of damp Okapa that still clings to them after 45 years.

My official boss in PNG was Frank Schofield, the Deputy Director of Research in the Department of Public Health. We normally kept in touch with each other through government channels but early in my stay he came to see me in the field. I was out in a remote village when I got his message, and he walked halfway along the path from the end of the road to meet me in a clearing in the forest, where we had our conference. He gave me complete freedom to follow my own plan of work, for which I was—and still am—very grateful. I am sorry that Frank was unable to join us at the conference in London.

I teamed up with Carleton Gajdusek to do many things, including the transmission experiments in chimpanzees. I performed the autopsies, which were done on kuru patients I had followed carefully from onset to death. I did each autopsy in the house where the patient died, assisted by close relatives and my team of assistants. My assistants were all young Fore men whom I had trained for the purpose and also taught to read and write. When we weren't waiting for someone to die we were walking in the forest or through village gardens to see patients. We became a close-knit team, and have remained life-long friends. I pay tribute here to the work of Mabage Ubenumu, Ove Yaraki, Tuniye Aboru, Awusa Mena, Auyana Winagaiya and Kene Nabu. Mabage later died of kuru, but I am delighted that his wife Kainamba (whom I also know well, from the village of Waisa) was able to celebrate the ‘end of kuru’ at the meeting in London (Mabage 2008). Tuniye and Kene have also died and Auyana and Ove were not well enough to travel to London for the meeting. Auyana assisted me with kuru surveillance for 30 years. Later, the field team changed a little and was strengthened by Anua Senavaiyo (see Anua 2008), Igana Alesagu and Kabina Yaragi, all of whom also worked with me for decades. Anua has died and we did not have sufficient funds, unfortunately, to bring Igana and Kabina to the meeting.

When the autopsies (limited to the brain) had been completed and the skin stitched back, the precious sterile samples for inoculation were taken to Okapa by road (walking or driving) and the next day to Lae by air charter. From Lae they were sent by air in dry ice to Melbourne, where Roy Simmons received and looked after them until he could send them on to the NIH. Roy was an invaluable colleague, with whom Carleton and I also collaborated on genetic studies in the kuru region. He played an essential role in getting the brain samples from the village to Bethesda. He died many years ago now, but his contribution has not been forgotten.

Not all the clinical descriptions of kuru matched in detail and I was keen to attract neurologist colleagues to make further independent clinical assessments of patients. This has been done recently by John Collinge and Dafydd Thomas (Collinge *et al.* 2008). Earlier, Stanley Prusiner worked with me in the field and saw patients at home in their village communities (Prusiner *et al.* 1982). In this context I wish to acknowledge, in particular, the contribution of Leonard Rail, who came to Okapa in the early days of kuru research to work with Carleton Gajdusek and performed electroencephalographic and electromyographic studies on kuru patients. Later, Len worked with me in the field. As well as clinical examinations we carried out cinema documentation of the patients. Len performed the examinations so I was free to be the cameraman. I had my Bolex camera, with 400 ft magazines, a battery-powered motor and a firm tripod, and we were able to get the best long sequences of kuru in the record. I am pleased to acknowledge Len's contribution to this and to our joint clinical assessment of kuru (Alpers & Rail 1971).

Initially, after the oral transmission of kuru through the local mortuary practices had been proposed (Alpers 1968), there was a strong opinion that oral transmission would not occur, which was seemingly supported by early experiments in which oral transmission to chimpanzees had—so far—proved negative. Accordingly, Stanley Prusiner and I devised an experiment in which hamsters dead from scrapie would be cannibalized by their cage-mates: transmission occurred, but at a variable take rate and with a wide range of incubation period, as predicted from the findings in kuru (Prusiner *et al.* 1985). Patricia Cochran carried out these experiments and I pay tribute to her meticulous work.

This selection of ‘tributes to research colleagues and other contributors to our knowledge about kuru’ has aimed to honour those—or, at least, some of those—whose contributions have not been acknowledged elsewhere in these proceedings. Moreover, it is a personal selection, based on my own experience, and has been further restricted for reasons of space. When we take into account those who were present at the meeting or have contributed to these Proceedings, and the selected group to whom tribute has been paid here, together with others—including, of course, the many patients suffering from kuru who have been examined and studied—it is clear that a large number of people, of all kinds—in the community, in government, in the laboratory, in the clinic—have been contributors, in their various ways, to our knowledge about kuru. I am happy here to pay tribute to their significant contributions.
